# The Collected Works of Sir Humphry Davy, Bart., LL.D. F.R.S

**Published:** 1839-10

**Authors:** 


Art. XVI.—*The Collected Works of Sir Humphry Davy, Bart.* LL.D. F.R.S. Edited by his brother, John Davy, M.D. F.R.S. Vol. I. *Memoirs of his Life.—London.* 1839. 8vo, pp. 475.

The warm affection of a brother has guided the pen of the author throughout this volume. Biography in such circumstances necessarily becomes eulogium. For every virtue of Davy, and for his immortal services to science, the gratitude and esteem of the public are not less than the admiration of his affectionate relative ; and if in this history of a great man by one who had just reason to be proud of him, fraternal partiality has sometimes given importance to trifles, and sometimes found excuses for foibles with which others only were offended, it is not for us to ascribe it as a fault of great magnitude in Dr. John Davy. Assuredly, we think many particulars of the early life of Davy might have been omitted without loss to the reader; and we should be disposed to extend this observation to nearly all the poetical fragments, which, although they bear the impress of Davy's elevated intellect, belong to that order of verse which no true lover of imaginative poetry reads but for the thoughts, or reads more than once. But readers of the highest order of intellect, and of the most exalted imagination, may and will peruse with deep gratification the fragments, expressed in prose, and taken from Davy's note-book, in which the great philosopher's habit of advancing his thoughts from the known and proved towards regions not yet opened to the distinct apprehension of human beings, is splendidly conspicuous. In contemplating the life and thoughts of men of this high class, whose clear vision, ranging from the vantage-ground of science, extends farther than that of ordinary men, we always feel desirous to question them of their ultimate views of themselves, and of the express and admirable universe around them. Few have thought so much on these high topics, and few have dared to be so candid as Davy : none ever arrived, by the paths of philosophy, at more noble and encouraging views of the progress and destiny of mankind, and of the vast unknown from which the dark curtain is not yet raised. If for the advantage alone of becoming acquainted with these, the innermost thoughts of a reflecting and powerful mind, we would strongly recommend every student to read the life of Davy. It will, however, be found both interesting and useful on other grounds. A clear, accurate, and succinct account is given of Sir Humphry's principal discoveries and writings; and several of his letters, all animated by the same spirit of investigation and enquiry, are interspersed with the details of his progress.

In all biographical reading, the most impressive, perhaps the most instructive, result is, that we see the whole drama of life, which in each individual's own case is so expanded as seldom to be contemplated as a whole, compressed into a small and appreciable space; the hopes of youth, the triumphs of manhood, and the yielding up of all to sure decay, perhaps to decrepitude and physical degradation. The decline of Davy was gradual. As in the case of Cuvier, Scott, and other great men, (however various their greatness,) the intellect survived much of the mere bodily power ; and, when the physical organs were more than ever bound to the earth, with which they were soon to be mixed, soared with even more earnest pinion towards another existence, into which they aspired to ascend. Believing his malady to be mortal, he rejected no human means of controlling or remedying it, making exertions, often of the most painful kind, to overcome the torpor of paralysis, which was but the beginning of death, and yet chiefly anxious to complete his intellectual labours. "I fight," said he, "against sickness and fate, believing I have still duties to perform, and that even my illness is connected in some way with my being made useful to my fellow-creatures." In such instances, the great gift of intellect is seen in all its value, and over it death has no victory. Like the dying gladiator (a subject consecrated alike by ancient art and modern poetry), the philosopher "consents to death, but conquers agony;" and, having combated, not for conquest, but for truth, passes through these worst humiliations of our mortal nature with a calmness, which truly gives to his final departure the air of a transition from this world of limited knowledge to a world of higher intellect, where more enlarged faculties will find a still more expanded field of exertion. Or if such thoughts be too presumptuous, it is still deeply interesting to watch the calm exit of an incomprehensible element of life, which has been exerted, in this short existence, for the never-ending benefit of all mankind.

We think the public under great obligations both to the editor and publisher for this beautiful and cheap edition of Sir Humphry Davy's complete works. It cannot fail to be patronised, as it well deserves to be, by every lover of science.

